# Alteration in gut mycobiota of patients with polycystic ovary syndrome

**DOI:** 10.1128/spectrum.02360-23

**Published:** 2023-09-13

**Authors:** Ke Chen, Huafeng Geng, Junbao Liu, Cong Ye

**Affiliations:** 1 Department of Gynecology, China-Japan Union Hospital of Jilin University, Changchun, Jilin, China; Nanjing Agricultural University, Nanjing, China

**Keywords:** polycystic ovary syndrome, gut mycobiota, *Saccharomyces*, *Lentinula*, *Aspergillus*

## Abstract

Polycystic ovary syndrome (PCOS) is a serious disease characterized by high androgen, insulin resistance (IR), hyperglycemia, and obesity, leading to infertility. The gut mycobiota has been reported to evolve in metabolic diseases including obesity, hyperglycemia, and fatty liver. However, little is known about the gut mycobiota and PCOS. In the current study, we recruited 17 PCOS patients and 17 age-matched healthy controls for community structure and functional analysis of the gut mycobiota. The results showed that PCOS patients have reduced diversity and richness of the gut microbiota compared with healthy controls. β-Diversity analysis showed that the community structure of the gut microbiota of patients with PCOS was significantly different from healthy controls. At the phylum level, PCOS patients have reduced *Basidiomycota* and increased *Ascomycota* compared with healthy controls. At the family level, the higher relative abundance of *Saccharomycetaceae* and lower *Trichosporonaceae* and *Ascomycota_unclassified* were detected in PCOS patients than in healthy controls. At the genus level, different microbial compositions were also observed between PCOS patients and healthy controls. In addition, PICRUSt2 showed that patients with PCOS have different microbial functions in the gut compared with healthy controls. LEfSe indicated that *Saccharomyces* and *Lentinula* were enriched in the fecal samples of PCOS patients, while *Aspergillus* was depleted compared with healthy controls. Our finding indicates that PCOS patients have different community structures and functions of the gut mycobiota, which provides new insight into PCOS pathogenesis and intervention.

**IMPORTANCE:**

It was found that intestinal fungi as well as serum metabolites in PCOS patients were significantly different from those in healthy subjects. However, no studies have been done to show exactly which fungus interacts with which bacteria in humans or which fungus acts alone. As fungal research progresses, it will be possible to fill this gap.

## INTRODUCTION

Polycystic ovary syndrome (PCOS) is a complex endocrine and metabolic abnormality common in women of childbearing age (1). It is characterized by chronic anovulation (disturbance or loss of ovulatory function) and hyperandrogenemia (excess production of male hormones in the woman’s body) (2). The main clinical manifestations are irregular menstrual cycle, infertility, hirsutism, and acne, which are the most common female endocrine disorders (3). The cause of PCOS has been suggested to be due to abnormal pubertal initiation, which may be called the “hyperpubertal” phenomenon (4). It has also been shown that the development of PCOS is not only associated with abnormalities in the hypothalamic-pituitary-ovarian axis and ovarian autocrine/paracrine regulation but also has a synergistic effect with extra-ovarian factors (5). In the meantime, insulin resistance, hyperinsulinemia, and obesity have all been shown to be extra-ovarian risk factors for PCOS (6). In addition, PCOS has a familial aggregation phenomenon. It is presumed to be a polygenic disease. In 2000, scholars investigated 139 PCOS patients and 137 control families and concluded that irregular menstruation in the mother, early baldness in the father, and hypertension in the father are independent genetic phenotypes that cause PCOS in daughters (7). Li Tan et al. showed that patients with PCOS may be associated with a family history of cardiovascular disease, with a greater influence on the mother (8). Environmentally, antiepileptic drugs, geography, nutrition, and lifestyle may be high risk factors for PCOS (9). In summary, the etiology of PCOS is caused by a combination of genetic and environmental factors (10). With the change in environmental factors and lifestyle, the incidence of PCOS has been increasing year by year in recent years (11). The prevalence of PCOS in women of childbearing age is approximately 4%–8% (2). The heterogeneous clinical presentation of PCOS not only severely affects the reproductive function of patients but also increases the incidence of estrogen-dependent tumors. The associated metabolic dysregulation includes hyperandrogenemia, insulin resistance, abnormal glucose metabolism, and abnormal lipid metabolism, leading to an increased incidence of type 2 diabetes and cardiovascular disease as well. In addition, approximately 70%–80% of patients with irregular menstruation and 85% of patients with anovulatory uterine bleeding have been reported to be due to PCOS (11).

The intestine provides an ideal habitat for the growth and proliferation of various microbial communities, including bacteria, fungi, and archaea, which are collectively referred to as intestinal microbes (12). The total number of gut microbial genes is about 150 times more than the number of genes in the human body. From the data, we can see that the intestinal flora is a very large group, so the intestinal bacteria are also called the “second genome” and “second brain” of the human body (12). There are 10^11^ intestinal microorganisms in the healthy human gut, of which about 0.1% are intestinal fungi (13). Gut microbiota has been reported to play a critical role in the development of many diseases (14 – 16). Recent studies demonstrated that the diversity and composition of the gut microbiota of PCOS patients changed (17). Recently, fungi have been found in feces, suggesting that colonization by intestinal fungi begins at birth. Compared with bacterial cells, fungal cells are large, complex, and have many unique metabolic pathways that play an important role in maintaining intestinal microecological homeostasis and responding to host immune responses (18). Therefore, the role of fungal flora in intestinal flora has been gradually discovered and emphasized. With the development of technologies such as high-throughput sequencing, more and more intestinal fungi are being detected and identified. Recent studies have identified the homeostasis of the intestinal fungal flora as a key factor affecting host health. Similar to intestinal bacteria, the role it plays in human health and disease cannot be ignored (19). As an important component of intestinal microecology, various components of the fungal cell wall (including beta-glucan, enzymatic glycans, mannans, chitosan, DNA, and RNA) can be recognized by host cells under physiological conditions, although at low levels (20). This activates innate and acquired immunity. This response inhibits the overgrowth of intestinal fungi or the colonization of exogenous pathogens. Similar to intestinal bacteria, dysbiosis of fungal flora can lead to immune dysregulation in the host, thereby causing disease (20). In a healthy state, intestinal commensal fungi and their hosts are in a reciprocal or paracrine symbiosis. The intestinal mucosal immune system can allow the presence of fungi, but the immune system activates protective mechanisms to regulate intestinal microecological homeostasis when the fungal flora is dysbiosis or pathogenic fungal infestation (21). The host immune system can also be activated by the bacterial flora to kill pathogenic fungi. Intestinal microecological homeostasis, but the over-proliferation of certain conditionally pathogenic fungi or infection by foreign pathogenic fungi when host immunity is reduced, can lead to severe dysbiosis of the host intestinal fungal flora, which can not only cause or exacerbate intestinal disease but also induce the development of diseases in organs other than the intestine (22).

Fungi can also act as separate members on the immunity of the organism. For example, dendritic cells promote Th17 responses when triggered by the Aspergillus fungus Aspergillus and the Rhizopus fungus Rhizopus (23). These studies have led to more interest being devoted to elucidating the relationship between intestinal fungi and disease. There have been a growing number of reports confirming that intestinal fungi are closely associated with inflammatory bowel disease. Mice with knockouts of the fungal pattern recognition receptors Dectin-1 and Dectin-3 were susceptible to inflammatory bowel disease (24). Further in these disease conditions, its genome was studied, and one of the fungi called *Candida tropicalis* was found to be significantly increased in abundance. Dectin-1 and Dectin-3 were also confirmed to be susceptible genes for inflammatory bowel disease by genome-wide association study assay (25). Other studies have found that fungi can induce colorectal tumorigenesis by modulating host immunity. This includes induction of inflammatory vesicle production, myeloid-derived suppressor cell differentiation, and interleukin-22 secretion by type 3 innate lymphoid cells (21). In addition to this, due to the large size of the fungus, it can bind competitively with bacteria in the intestinal tract and inhibit the proliferation of probiotics, thereby promoting tumorigenesis and development. Besides, due to the large size of the fungus, it can competitively bind with bacteria in the intestine and inhibit the proliferation of probiotic bacteria, thus promoting the development and progression of tumors (26). Besides being associated with the intestinal tract, fungi are also closely associated with many pulmonary diseases (27). In allergic bronchopulmonary aspergillosis, *Aspergillus fumigatus* can induce the activation of RelB, a non-classical pathway of the nuclear factor kappa B (NF-κB) pathway, through Card9, leading to the secretion of interleukin-5 and the induction of a Th2-type immune response (20). This means that fungi and asthma diseases of the respiratory tract are closely related. To investigate whether intestinal fungi are associated with PCOS and whether there is a “gut-ovarian axis” pathway, a previous study evaluated the potential of microbiologically relevant diagnostic methods for PCOS by examining the intestinal fungal and serum metabolomic profiles of PCOS patients (28). It was found that intestinal fungi as well as serum metabolites in PCOS patients were significantly different from those in healthy subjects. However, no studies have been done to show exactly which fungus interacts with which bacteria in humans or which fungus acts alone. As fungal research progresses, it will be possible to fill this gap.

## RESULTS

### Descriptive statistics

A total of 17 PCOS patients and 17 healthy controls were recruited in this study. The average age of PCOS patients is 25.53 years, while the average age of healthy controls is 23.87 years, but no significance was identified (Table 1). Consistently, no significance was observed in height between PCOS and healthy controls (Table 1). However, higher body weight was detected in PCOS patients than in healthy controls (*P* = 0.0490, Table 1). Similarly, PCOS patients have increased body mass index (BMI) compared with the healthy controls (*P* = 0.0665, Table 1). The serum hormone levels of PCOS patients, including luteinizing hormone (LH), follicle-stimulating hormone (FSH), prolactin (PRL), estradiol (E2), testosterone (T), fasting blood glucose (FBG), total cholesterol (TCHO), triglyceride (TG), low-density lipoprotein (LDL), high-density lipoprotein (HDL), and fasting insulin (FSlns), were also determined for PCOS diagnosis (Table 1).

**TABLE 1:** Characteristics of study individuals^*a*^

Characteristics	Healthy controls (*n* = 17)	Patients with PCOS (*n* = 17)	*P* value
Demographics			
Mean age (years), mean ± SD	23.83 ± 2.758	26.14 ± 4.383	0.253
Height, cm, mean ± SD	162.5 ± 4.642	160.5 ± 4.274	0.1946
Weight, kg, mean ± SD	54.10 ± 3.178	65.21 ± 10.17	0.0011
BMI, mean ± SD	20.51 ± 1.325	25.60 ± 4.634	0.0019
Disease characteristics			
LH	NA	9.18 ± 3.882	
FSH	NA	5.236 ± 0.6058	
PRL	NA	256.2 ± 223.4	
E2	NA	87.11 ± 48.63	
T	NA	0.6767 ± 0.3164	
FBG	NA	5.002 ± 0.6364	
TCHO	NA	5.203 ± 0.7693	
TG	NA	1.765 ± 1.461	
LDL	NA	3.043 ± 0.4201	
HDL	NA	1.328 ± 0.3870	
FSlns	NA	17.05 ± 14.36	

^*a*^ LH, luteinizing hormone; FSH, follicle-stimulating hormone; PRL, prolactin; E2, estradiol; T, testosterone; FBG, fasting blood glucose; TCHO, total cholesterol; TG, triglyceride; LDL, low-density lipoprotein; HDL, high-density lipoprotein; FSlns, fasting insulin.

### Alpha diversity of the gut mycobiota

**Fig 1:**
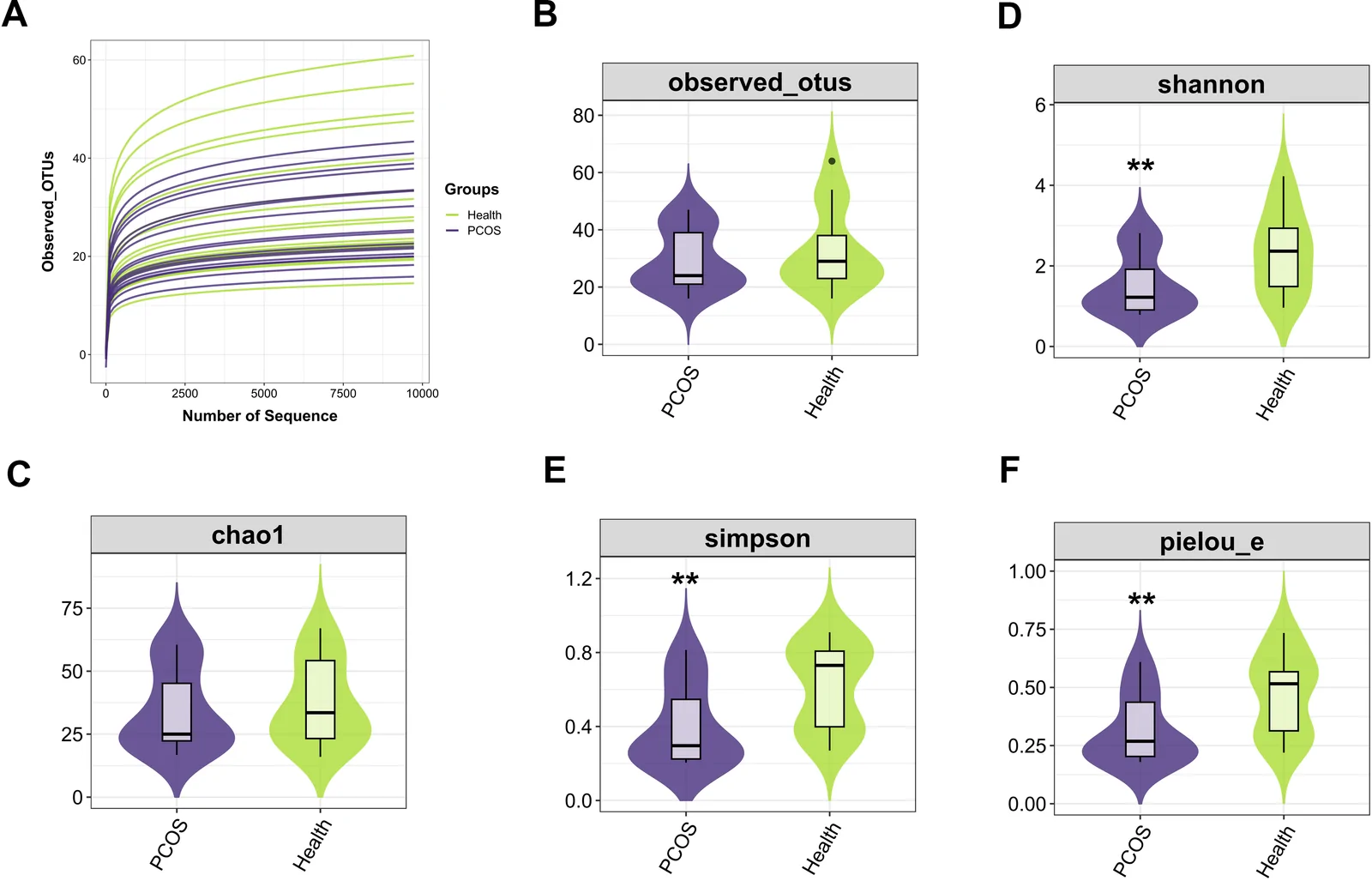
Alpha diversity of the gut mycobiota. Alpha diversity analysis showed gut mycobial richness, diversity, and evenness by OTUs, Shannon, Chao 1, Simpson, and pielou_e. OTU index was induced in PCOS patients compared with healthy individuals by Mann-Whitney *U*-test, *n* = 17 (**A and B**). Chao 1 also showed that the gut mycobial richness decreased in fecal samples of PCOS patients (**D**). Besides, Shannon (**C**), Simpson (**E**), and pielou_e (**F**) results indicated that all indexes are reduced obviously in the fecal samples of PCOS patients. All data were based on Mann-Whitney *U*-test, *n* = 17. **P < 0.01 indicates significance.

Alpha diversity analysis was performed to detect the diversity, richness, and evenness of gut mycobiota. We first showed that observed operational taxonomic units (OTUs) of gut mycobiota did not significantly differ between the PCOS group and the healthy group (Mann-Whitney *U*-test, Fig. 1A and B). Similarly, the Chao 1 index has no significant difference between groups (Mann-Whitney *U*-test, Fig. 1C). Decreased Shannon and Simpson indices were observed in patients with PCOS compared with those in the healthy group, indicating that patients with PCOS had reduced mycobiota diversity in the fecal samples (*P* < 0.01, Mann-Whitney *U*-test, Fig. 1D and E). Next, we further confirmed the reduced fecal mycobiota diversity in individuals with PCOS by the pielou_e index (*P* < 0.01, Mann-Whitney *U*-test, Fig. 1F). Collectively, these results suggest that PCOS significantly reduced the richness and diversity of gut mycobiota in patients.

### Microbial structure by beta-diversity analysis

**Fig 2:**
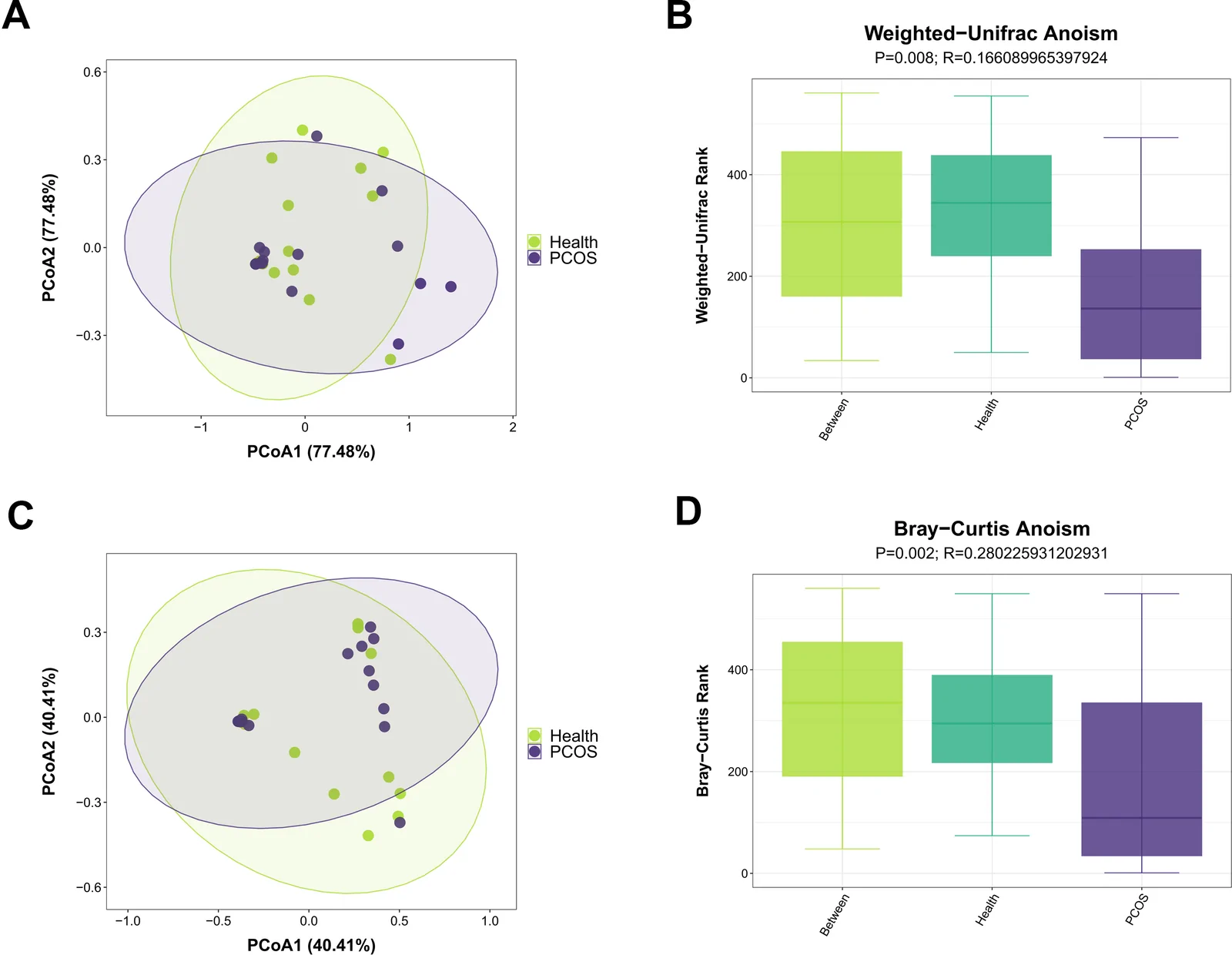
Microbial structure by beta-diversity analysis. (**A**) PCoA and beta-diversity analyses were performed; using weighted Unifrac distance we separated the PCOS patients and healthy group (*n* = 17). (**B**) ANOSIM analysis results showed that the microbial structure was different between PCOS patients and healthy individuals (*P* = 0.008, *R* = 0.1661, ANOSIM, *n* = 17). (**C**) PCoA based on Bray-Curtis distance showed that the gut mycobiota of PCOS patients was different from the healthy group (*P* = 0.002, *R* = 0.2802, ANOSIM, *n* = 17).

To explore differences in gut mycobiota structure in all 34 fecal samples, beta-diversity analysis was performed. We found that gut community of PCOS group was different from those of the healthy group, which is shown by principal coordinate analysis (PCoA) based on unweighted Unifrac distance (Fig. 2A). Then, analysis of similarities (ANOSIM) revealed significant differences in gut fungus between healthy controls and PCOS patients (*P* = 0.008, *R* = 0.1661, Fig. 2B). To further confirm these results, PCoA based on Bray-Curtis distance was used. Similarly, the gut mycobiota in the PCOS patients was significantly different from those in the healthy group (*P* = 0.002, *R* = 0.2802, ANOSIM, Fig. 2C and D). Altogether, these findings imply that gut mycobiota structure is significantly different between the healthy group and the PCOS group.

### Fecal microbial community composition

**Fig 3:**
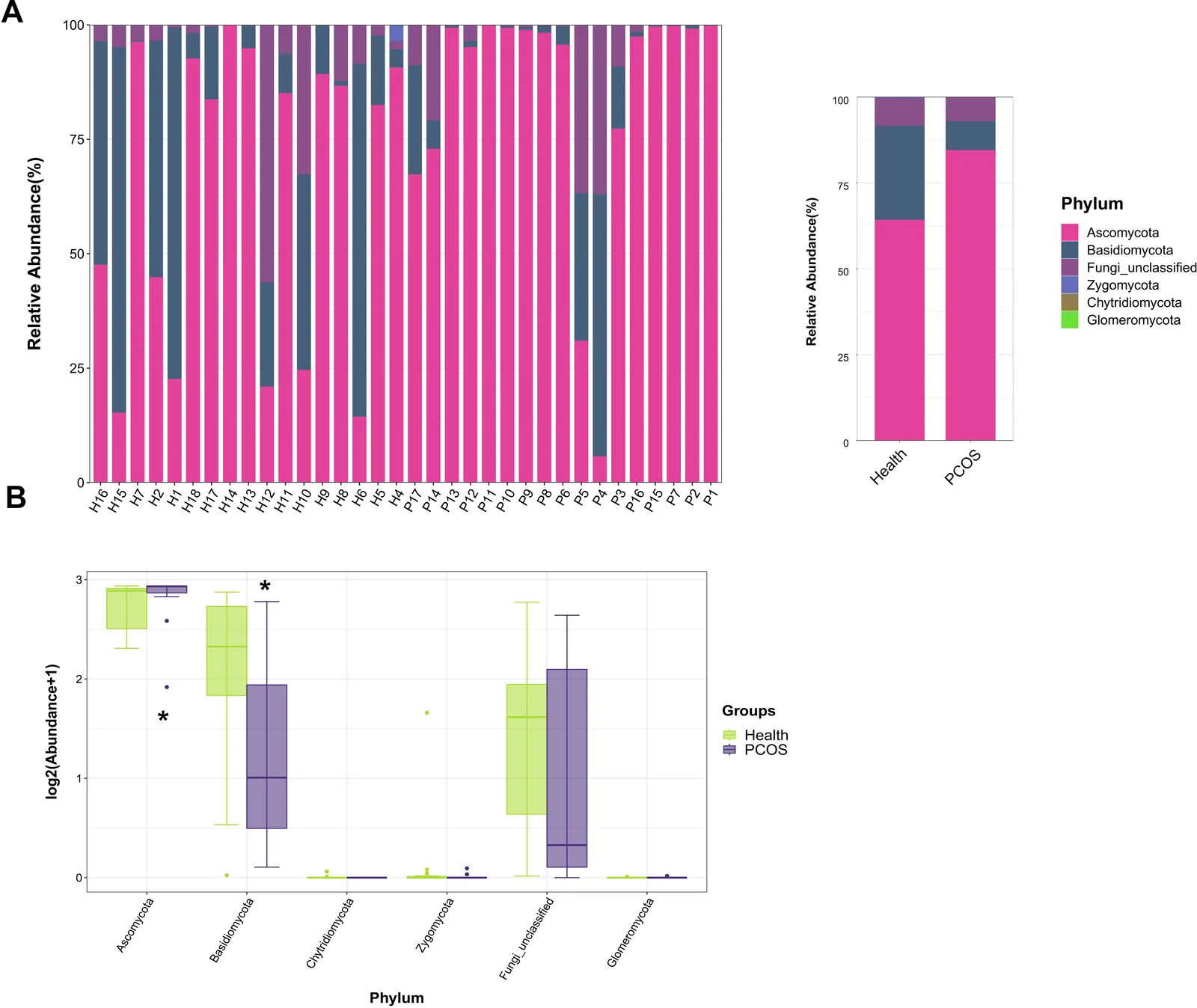
Fecal microbial community composition at the phylum level. (**A**) At the phylum level, the fecal mycobiota community of PCOS patients and healthy individuals had a significant difference. (**B**) Mann-Whitney *U*-test was performed followed by FDR correction (*n* = 17), *P < 0.05 indicates significance.

We then investigated the composition of gut mycoflora in all healthy and PCOS patients. At the phylum level, gut mycobiota composition between the PCOS group and healthy group was different, which was characterized by an increased relative abundance of *Ascomycota* and reduced *Basidiomycota* and *Fungi_unclassified* in PCOS patients relative to healthy controls (Fig. 3A). To confirm these results, Mann-Whitney *U*-test was performed followed by false discovery rate (FDR) correction. We showed that patients with PCOS had a higher relative abundance of *Ascomycota* than healthy controls (*p_fdr_* = 0.014, Mann-Whitney *U*-test, Fig. 3B), while PCOS patients had lower relative abundances of *Basidiomycota* than those in the healthy group (*p_fdr_* = 0.014, Mann-Whitney *U*-test, Fig. 3B).

**Fig 4:**
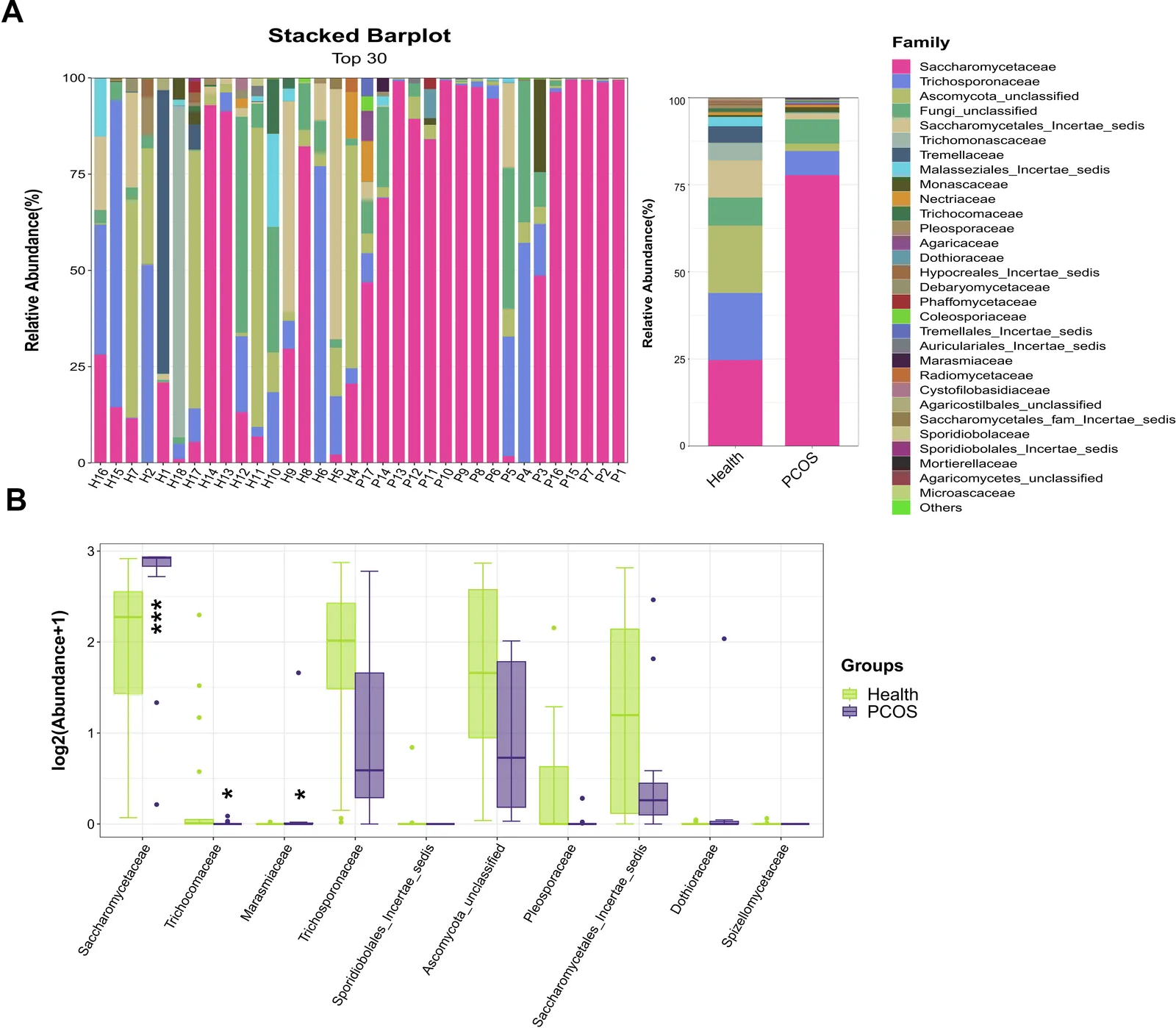
Fecal microbial community composition at the family level. (**A**) At the family level, fecal mycobiota community composition had a great difference between PCOS patients and healthy individuals. (**B**) The top 10 different genera were analyzed by Mann-Whitney *U*-test (*n* = 17). *P < 0.05 and ***P < 0.001 indicate significance.

At the family level, enhanced relative abundances of *Saccharomycetaceae, Monascaceae, Agaricaceae,* and *Dothioraceae* were detected in the fecal sample of PCOS patients than those in the healthy controls (Fig. 4A), while reduced relative abundances of *Trichosporonaceae, Ascomycota_unclassified, Saccharomycetales_Incertae_sedis, Trichomonascaceae, Tremellaceae, Malasseziales_Incertae_sedis,* and *Debaryomycetaceae* were observed in the fecal sample of PCOS patients than in the healthy controls (Fig. 4A). Next, we examined the top 10 different families by Mann-Whitney *U*-test. These results showed that the relative abundances of *Saccharomycetaceae* (*p*_fdr_<0.001) and *Marasmiaceae* (*p*_fdr_ = 0.048) were higher in patients with PCOS than in the healthy controls, while *Trichocomaceae* (*p*_fdr_ = 0.021) was higher in the healthy group than the PCOS group (Fig. 4B).

**Fig 5:**
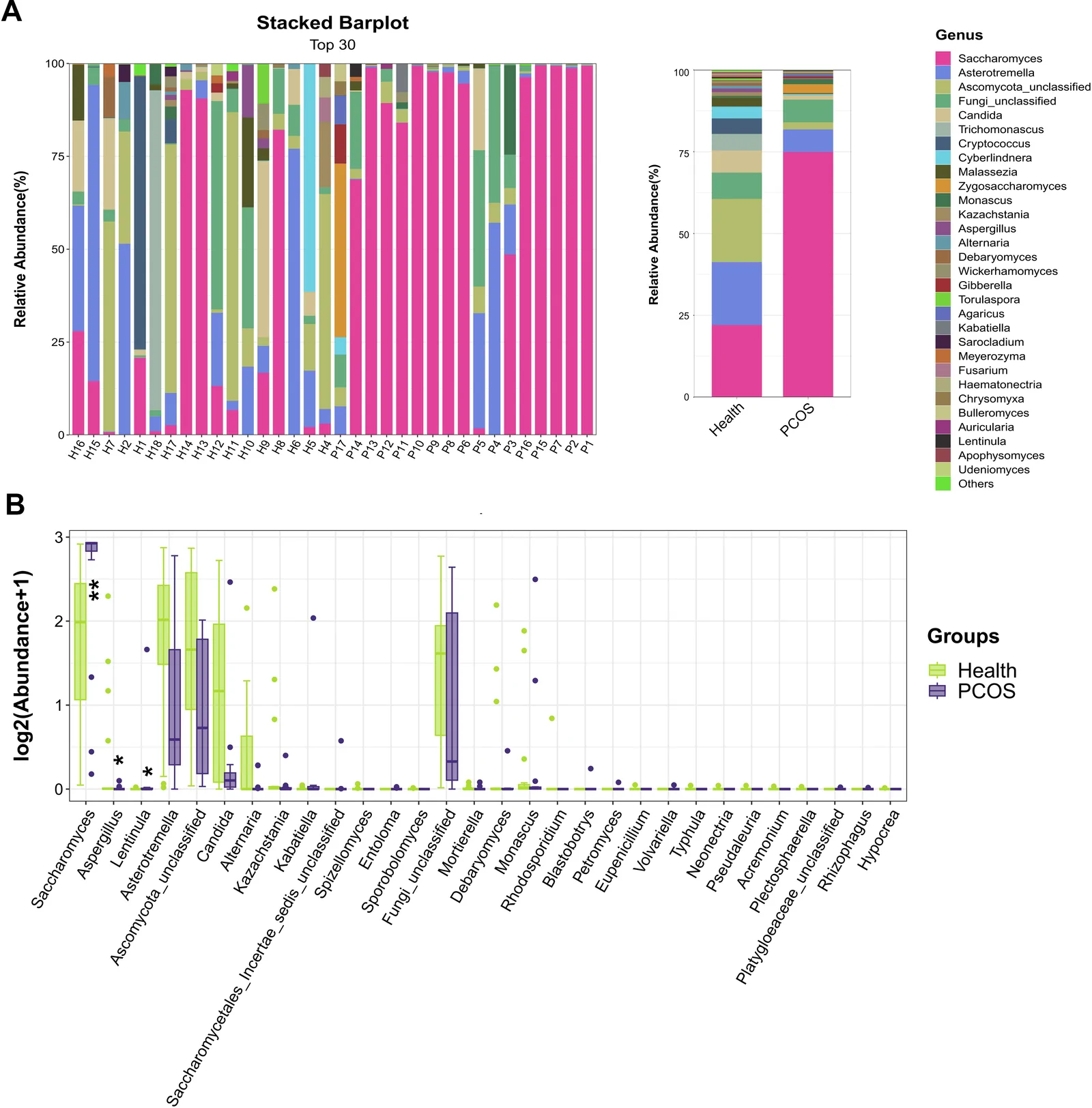
Fecal microbial community composition at the genus level. (**A**) At the genus level, there was an obvious difference between PCOS patients and the healthy group in the fecal mycobiota community composition (*n* = 17). (**B**) The top 30 different genera were confirmed by Mann-Whitney *U-*test (*n* = 17).*P < 0.05 and **P < 0.01 indicate significance.

At the genus level, higher relative abundances of *Saccharomyces, Candida, Zygosaccharomyces, Monascus, Gibberella, Agaricus,* and *Kabatiella* were identified in PCOS patients than in healthy controls (Fig. 5A), while lower *Asterotremella, Ascomycota_unclassified, Trichomonascus, Cryptococcus, Cyberlindnera, Malassezia, Kazachstania, Aspergillus, Alternaria, Debaryomyces, Wickerhamomyces, Torulaspora,* and *Sarocladium* were observed in PCOS patients than in healthy controls (Fig. 5A). Moreover, the top 30 different genera were identified by Mann-Whitney *U*-test. We found that *Saccharomyces* (*p*_fdr_ = 0.0011) was greater in the PCOS group than in the healthy group (Fig. 5B), while *Aspergillus* (*p*_fdr_ = 0.046) and *Lentinula* (*p*_fdr_ = 0.048) were lower in the PCOS group than in the healthy group. Taken together, these results suggest that PCOS patients had changed the gut mycobiota composition.

### Microbial function predication

**Fig 6:**
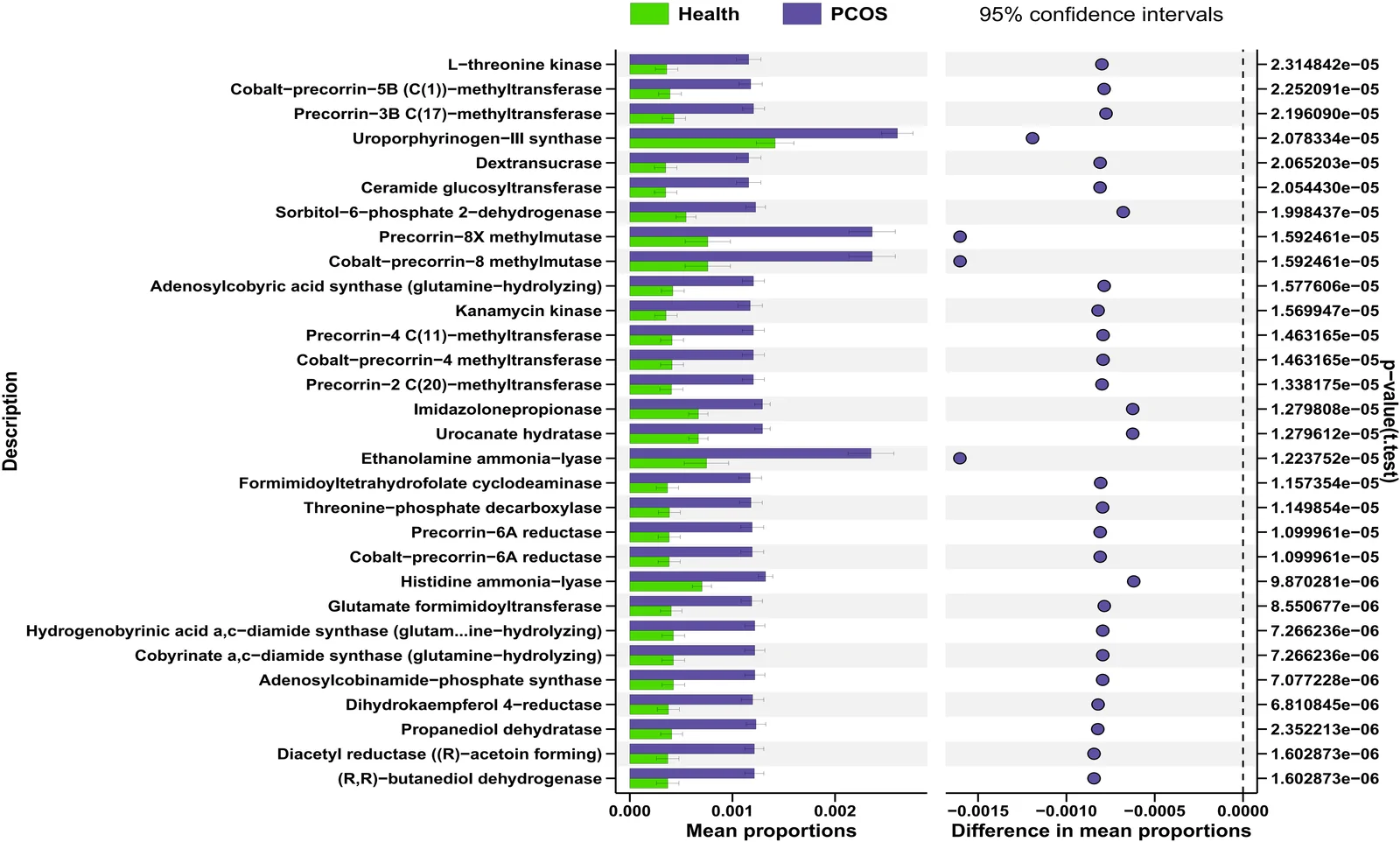
Microbial functions predication using PICRUSt2.

We further studied the top 30 gut mycoflora functions of fecal samples using PICRUSt2. The results showed that 30 mycobiota functions, including L-threonine kinase (*p*_fdr_ <0.001), cobalt-precorrin-5B (C(1))-methyltransferase (*p*_fdr_ <0.001), precorrin-3B C(17)-methyltransferase (*p*_fdr_ <0.001), uroporphyrinogen-III synthase (*p*_fdr_ <0.001), dextransucrase (*p*_fdr_ <0.001), ceramide glucosyltransferase (*p*_fdr_ <0.001), sorbitol-6-phosphate 2-dehydrogenase (*p*_fdr_ <0.001), and cobalt-precorrin-8 methylmutase (*p*_fdr_ <0.001), were significantly enriched in the gut mycobiota of PCOS group compared with the healthy group (Fig. 6). These results indicate that PCOS patients had altered gut mycobiota functions.

### PCOS patient-associated microbial species

**Fig 7:**
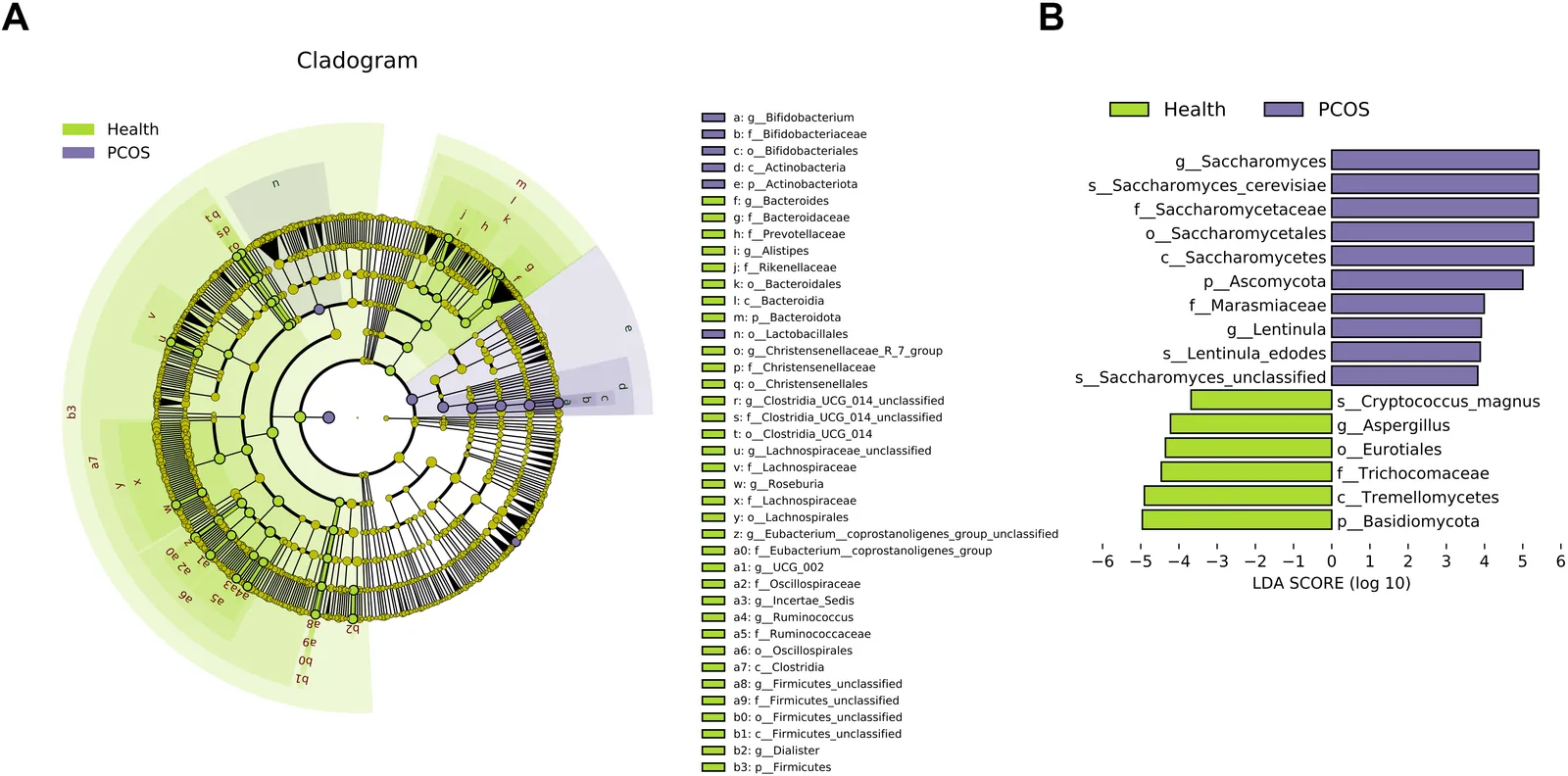
PCOS patient-associated microbial species. (**A and B**) Different microbial genera were identified by LEfSe [LDA score (log 10) ＞4].

To identify the different taxa enriched in each group, linear discriminant analysis (LDA) effect size (LEfSe) was performed [LDA score (log 10) >4]. We revealed that *Saccharomyces* and *Lentinula* were greater in the fecal samples of the PCOS group than in the healthy group (Fig. 7A and B), while *Aspergillus* was depleted in the fecal samples of the PCOS group compared with the healthy group (Fig. 7A and B). Collectively, these results suggest that PCOS patients had altered gut microbial species.

## DISCUSSION

PCOS is a heterogeneous disease associated with endocrine and reproductive disorders in women. It affects females of 18–44 age and is the most common endocrinopathy affecting reproductive-aged women (29). It manifests in a wide range of symptoms, including (but not limited to) hirsutism, amenorrhea, oligomenorrhea, obesity, acne vulgaris, infertility, alopecia, and insulin resistance (30). Studies showed that changes in levels of sex steroids affect the composition of the gut microbiota (31), and PCOS characterized by higher androgen also leads to a decrease in the diversity and composition of gut microbiota (32). At present, the research on gut microbiota mainly focuses on bacteria, and the research on gut fungi is relatively few. It is worth noting that fungal members of the gut microbiota have been shown to influence host immune responses by inhibiting or promoting local inflammatory responses (33). Similar to the bacteria, dysbiosis of fungal flora can also lead to host immune dysregulation, which can cause diseases. Several fungi were found to be associated with liver injury, lipid metabolism, and the development of non-alcoholic fatty liver disease (34). Fungi were also proven to participate in the dysbiosis of gut microbiota in patients with primary sclerosing cholangitis (35). Therefore, we examined the gut fungal flora in PCOS patients. In this study, we found that the gut fungal flora structure and function were altered in patients with PCOS in northeast China. Similar to our findings, previous studies have found reduced alpha diversity and altered β-diversity in the gut microbiota of PCOS patients compared to healthy controls.

Through the analysis of community composition, we found that *Saccharomyces* was enriched in the gut of PCOS patients. Notably, a similar situation occurs in the gut of depressed patients (36), chronic kidney disease (37), and *Clostridioides difficile* infection (38). The enrichment of *Saccharomyces* can trigger an immune reaction in the intestinal mucosa. In addition, the colonization of a large number of *Saccharomyces cerevisiae* in the intestine increases intestinal permeability, affects the intestinal barrier and host metabolism, and worsens colitis (39). *Saccharomyces boulardii (Sb*) can upregulate serotonin transporter (SERT) by epidermal growth factor receptor (EGFR) activation and modulate gut microbiota to inhibit the gut motility (40). Its colonization in the gut is likely to affect intestinal peristalsis and cause diseases. The high level of *Saccharomyces* was responsible for the high concentration of serum-free light chain (FLC) κ in chronic kidney disease (CKD) patients. As FLCs, such as FLC κ, accumulate in the circulation, their concentrations progressively increase with renal damage in CKD patients (37). This may suggest the role of *Saccharomyces* in PCOS, but its specific mechanism still needs to be further explored.

Furthermore, *Lentinula* was found to be enriched in PCOS patients. Its main metabolite *L. edodes* biologically active polysaccharides is a beta-glucan recognized by a variety of cell surface receptors including monocytes/macrophages, neutrophils, natural killer (NK) cells, and T and B cells (41). The interaction of polysaccharides with CR3 (expressed on neutrophils, monocytes, macrophages, and NK cells) (42) leads to enhanced spleen tyrosine kinase (Syk) phosphorylation, augmented phosphatidylinositol3 kinase (PI3K) activation, and stimulation of NF-κB leading to the cytokine production, including interleukin (IL)−2, IL-10, and tumor necrosis factor alpha (43). When the body is in a healthy state, these changes will regulate the body’s immune response and improve immunity, but in PCOS patients, the over-enrichment of *Lentinula* may become the main cause of inflammation. Therefore, analyzing and studying microbial metabolites are likely to be important breakthroughs in the treatment and prevention of PCOS.

These results showed that *Aspergillus* was significantly reduced in patients with PCOS. A similar situation occurs in patients with rheumatoid arthritis (44). This has been demonstrated in previous studies that there is a negative association between *Aspergillus* and Huntington’s disease risk (45). *Aspergillus* is an effective producer of β-galactosidase (46), which can inhibit colorectal cancer and increase the abundance of probiotics of *Bifidobacterium* and *Lactobacillus* (47). In addition, the fungus *Aspergillus terreus* was rich in a characteristic metabolite—butenolide—which has a wide range of biologic activities, such as anti-inflammatory, antioxidant, α-glucosidase inhibitor activities, and it may improve type 2 diabetes (48). This may indicate that the effects of PCOS on body health may be produced by changing the gut fungal composition and the content of its metabolites. Further studies on *Aspergillus* species will help to understand their role in PCOS fecal fungal communities and their implications for human health.

In summary, by comparing the gut fungi between PCOS patients and healthy controls, we found that there were significant differences in the richness and species diversity of gut fungi in PCOS patients. Changes in fungal communities indicate significant dysbiosis between healthy and PCOS patients. Among them, there is a significant difference in abundance among *Saccharomyces*, *Lentinula,* and *Aspergillus*. At the same time, changes in the abundance of these related fungi may play a momentous role in systemic diseases. And its specific mechanism still needs further exploration. This will provide new research ideas and strategies for the treatment and prevention of PCOS through fungal communities.

## MATERIALS AND METHODS

### Human sample collection

The study was approved by the Ethics Committee of Jilin University according to the Council for International Organizations of Medical Sciences. All participants were recruited from the China Japan Union Hospital between December 2022 and June 2023. We recruited 17 patients with PCOS and 17 healthy controls of Chinese ancestry. Written informed consent was obtained from all participants. Women with PCOS were diagnosed according to the 2003 Rotterdam criteria and previous study (17). All patients with PCOS had not received PCOS-related treatment. The healthy controls had regular menstrual cycles, normal ovarian morphology, and normal levels of hormones. Height, body weight, and BMI were detected. Peripheral blood samples were collected from all subjects during days 2–4 of spontaneous cycles after an overnight fast. Fecal samples from PCOS and healthy individuals were collected for 16S rRNA sequence.

### Hormone-level determination

Concentrations of serum hormones, including FSH and LH, were tested by radioimmunoassay as previously described (17). The levels of estradiol, testosterone, and androstenedione were measured using liquid chromatography-mass spectrometry (Sciex Triple Quad 6500+). The levels of fasting serum glucose, serum insulin, triglycerides, total cholesterol, high-density lipoprotein cholesterol, and low-density lipoprotein cholesterol were measured using an autoanalyzer (Beckman Coulter AU5800).

### Fecal DNA extraction

DNA from different samples was extracted using the CTAB according to the manufacturer’s instructions. The reagent which was designed to uncover DNA from trace amounts of sample has been shown to be effective for the preparation of DNA of most bacteria. Nuclear-free water was used for blank. The total DNA was eluted in 50 µL of Elution buffer and stored at −80°C until measurement in the PCR by LC-Bio Technology Co., Ltd (Hang Zhou, China).

### PCR amplification and ITS sequencing

The primer used for this study is F(5′-GAACCWGCGGARGGATCA-3′) and R(5′-GCTGCGTTCTTCATCGATGC-3′). The 5*'* ends of the primers were tagged with specific barcodes per sample and sequencing universal primers. PCR amplification was performed in a total volume of 25 µL reaction mixture containing 25 ng of template DNA, 12.5 µL PCR Premix, 2.5 µL of each primer, and PCR grade water to adjust the volume. The PCR conditions to amplify the ITS fragments consisted of an initial denaturation at 98°C for 30 s; 32 cycles of denaturation at 98°C for 10 s, annealing at 54°C for 30 s, and extension at 72°C for 45 s; and then final extension at 72°C for 10 min. The PCR products were confirmed with 2% agarose gel electrophoresis. Throughout the DNA extraction process, ultrapure water, instead of a sample solution, was used to exclude the possibility of false-positive PCR results as a negative control. The PCR products were purified by AMPure XT beads (Beckman Coulter Genomics, Danvers, MA, USA) and quantified by Qubit (Invitrogen, USA). The amplicon pools were prepared for sequencing, and the size and quantity of the amplicon library were assessed on Agilent 2100 Bioanalyzer (Agilent, USA) and with the Library Quantification Kit for Illumina (Kapa Biosciences, Woburn, MA, USA), respectively. The libraries were sequenced on the NovaSeq PE250 platform.

### Statistical analyses and visualization

Samples were sequenced on an Illumina NovaSeq platform according to the manufacturer’s recommendations, provided by LC-Bio. Paired-end reads were assigned to samples based on their unique barcode and truncated by cutting off the barcode and primer sequence. Paired-end reads were merged using Pear. Quality filtering on the raw reads was performed under specific filtering conditions to obtain the high-quality clean tags according to the fqtrim (v0.94). Chimeric sequences were filtered using Vsearch software (v2.3.4). After dereplication using DADA2, we obtained feature table and feature sequence. Alpha diversity and β-diversity were calculated by QIIME2, in which the same number of sequences was extracted randomly by reducing the number of sequences to the minimum of some samples, and the relative abundance (X fungicount/total count) is used in fungi taxonomy. Alpha diversity and β-diversity were analyzed by QIIME2 process, and pictures were drawn by R (v3.5.2). The sequence alignment of species annotation was performed by the QIIME2 plugin feature-classifier, and the alignment databases were RDP and Unite.

## Data Availability

The raw data supporting the conclusion of this article will be made available by the authors, without undue reservation, to any qualified researcher.
